# Reduction in regulatory T cells in preterm newborns is associated with necrotizing enterocolitis

**DOI:** 10.1038/s41390-023-02658-3

**Published:** 2023-06-21

**Authors:** Ilenia Pacella, Maria Di Chiara, Rita Prota, Chiara De Luca, Annalisa Cardillo, Elena Potenza, Alessandra Pinzon Grimaldos, Valeria Pinna, Silvia Piconese, Gianluca Terrin

**Affiliations:** 1 Department of Internal Clinical Sciences, Anesthesiology and Cardiovascular Sciences, Sapienza University of Rome, Rome, Italy; 2 Department Maternal Infantile and Urological Sciences, Sapienza University of Rome, Rome, Italy; 3 Neuroimmunology Unit, IRCCS Fondazione Santa Lucia, Rome, Italy; 4 Laboratory affiliated to Istituto Pasteur Italia – Fondazione Cenci Bolognetti, Rome, Italy

## Abstract

**Background:**

Despite multifactorial pathogenesis, dysregulation of inflammatory immune response may play a crucial role in necrotizing enterocolitis (NEC). Regulatory T cells (Tregs) are involved in immune tolerance early in life. We aimed to investigate the predicting role of Tregs in developing NEC in neonates at high risk.

**Methods:**

We studied six newborns with a diagnosis of NEC (cases) in comparison with 52 controls (without NEC). We further classified controls as neonates with feeding intolerance (FI) and neonates without it (FeedTol). The rate of female and male neonates (sex defined as a biological attribute) was similar. We analyzed the blood frequency of Tregs (not overall numbers) at three time points: 0–3 (T0), 7–10 (T1), and 27–30 (T2) days after birth by flow cytometry. Neonates’ sex was defined based on the inspection of external genitalia at birth.

**Results:**

We observed, at T0, a significantly lower frequency of Tregs in NEC cases (*p* < 0.001) compared with both FI (*p* < 0.01) and FeedTol controls (*p* < 0.01). Multivariate analysis reported that the occurrence of NEC was independently influenced by Treg frequency at birth (*ß* 2.98; *p* = 0.039).

**Conclusion:**

Tregs frequency and features in the peripheral blood of preterm neonates, early in life, may contribute to identifying neonates at high risk of developing NEC.

**Impact:**

- Regulatory T cells may play a pivotal role in regulating the immune response in early life. Reduction of Tregs in early life could predispose preterm newborns to necrotizing enterocolitis.
- Early markers of necrotizing enterocolitis are still lacking. We demonstrated a predicting role of assessment of regulatory T cells in the diagnosis of this gastrointestinal emergency.
- Early identification of newborns at high risk of necrotizing enterocolitis through measurement of regulatory T cells may guide clinicians in the management of preterm newborns in order to reduce the development of this severe condition.

## Introduction

Necrotizing enterocolitis (NEC) is an inflammatory disease of the intestine that represents the major gastrointestinal emergency in preterm neonates.^1^ NEC remains a leading cause of morbidity and mortality in preterm infants, and it is the first cause of short-bowel disease syndrome.^2^ The risk of NEC occurrence seems to be higher in male other than female neonates.^3^ Despite advances in clinical care and medical technology that have improved the ability to support premature infants, the prevalence of NEC has not decreased.^4^ A prompt diagnosis of NEC may significantly influence the long-term prognosis of affected newborns.^1^ Despite prematurity being a major risk factor for the occurrence of NEC, early identification of those newborns at high risk of developing the disease is still a challenge for neonatologists. Furthermore, many preterm neonates present a self-limiting benign clinical condition, defined as feeding intolerance (FI). It is widely agreed that FI represents a temporary clinical manifestation that may overlap with that of an impending NEC.^5^

Despite its pathogenesis is multifactorial, the immune system plays a crucial role in the development of NEC. The susceptibility to NEC might rely on a developmental process involving immature intestinal integrity and immune dysregulation.^4^ In particular, the limitation of inflammatory response is essential to control intestinal inflammation and its consequences. We herein hypothesized that regulatory T cells (Tregs), limiting inflammation and ensuring immunologic tolerance, may play a pivotal role in pathways of NEC.^6^ Tregs are a sub-population of T lymphocytes specialized in the maintenance of immune homeostasis through their predominant suppressive effect on many types of immune cells.^7,8^ Tregs, identified as CD4^+^CD25^high^ cells, are characterized by the expression of the transcription factor fork head box protein 3 (FOXP3), an essential factor for Treg development and function.^9,10^ Human Tregs are also characterized by the low expression of CD127.^11^ In the last decade, it has been established that human Tregs are a heterogeneous cell subset in which three functionally and phenotypically different subpopulations can be distinguished based on their expression of CD45RA and FOXP3: non-Tregs (CD45RA^-^FOXP3^low^), resting Tregs (CD45RA^+^FOXP3^low^) and activated Tregs (CD45RA^-^FOXP3^high^).^12^ Functional analysis demonstrated that both activated (hereafter actTreg) and resting Tregs are potently suppressive in vitro, and once stimulated resting Tregs differentiate into actTregs and proliferate in vitro and in vivo.^12,13^

Recent studies, performed in a neonatal mouse and rat model, have reported a delayed migration and a delayed ontogeny of Tregs in the intestinal tract and a reduced proportion of Foxp3+ Tregs in the intestinal mucosa.^14,15^ In humans, available evidence reported a reduced frequency of Tregs associated with confirmed NEC.^6,16,17^ In particular, patients with the active disease showed a reduction of Tregs as epiphenomenon of intestinal inflammation.^16^ However, there is no study evaluating the predisposing role of Tregs on the occurrence of NEC disease in preterm neonates.

We aimed to investigate the role and function of Tregs in early life in newborns at risk of NEC disease.

## Materials and methods

### Study design and population

We designed an observational study enrolling preterm newborns consecutively admitted into the Neonatal Intensive Care Unit (NICU) of Policlinico Umberto I, Sapienza University of Rome, between November 2020 and November 2021. We excluded neonates with major congenital malformations (including ambiguous genitalia), inborn errors of metabolism, congenital infections, intraventricular hemorrhage stage ≥3, death or transfer to another hospital before 72 h of life, and incomplete clinical data.

Clinical data were prospectively recorded for all enrolled neonates during the hospital stay. Whole peripheral blood (100 μl) from each neonate was collected in EDTA-coated Microvette® tubes (Sarstedt AG Co, Numbrecht, Germany) by heel stick, avoiding contamination with dust, at three time points: 0–3 (T0), 7–10 (T1), and 27–30 (T2) days after birth. Blood samples were codified until the statistical analysis.

Among eligible subjects, we classified neonates as cases or controls according to the appearance of specific signs and symptoms of NEC at any time during the hospital stay. In particular, diagnosis of NEC (Bell Stage II) was posed in the presence of biliary or bloody gastric residual, bloody stools associated with systemic symptoms (i.e., poor perfusion, muscle hypotonia or hypertonia, lethargy, progressive increase in O_2_ requirement, bradycardia, unstable body temperature, unexplained and persistent metabolic acidosis, unexplained and persistent hyperglycemia) and/or with radiological signs of NEC.^5,18^

Thus, we classified as cases newborns with a diagnosis of NEC stage II, and as controls newborns without signs and symptoms of NEC, during the entire period of hospital stay. NEC diagnosis was posed by a researcher blinded to study aims. The classification in cases and controls was made independently of Treg results. Researchers performing Tregs analysis were blinded to neonates’ clinical conditions.

Staging of NEC was established according to Bell Stage criteria and was confirmed after an agreement between three researchers.^19^ Three researchers confirmed patients’ classification in Case and Control Groups. Among Control Group, we also classified newborns with the occurrence of FI defined by the presence of gastric residuals >50% of the previous feed given by enteral nutrition associated with the need for parenteral nutrition (PN) for more than 10 days of life.

### Flow cytometry

To characterize the Treg population in neonates, a multicolor flow cytometry experiment was performed in whole blood samples, freshly collected at each time point, as follows: first, 1 μl of undiluted Fixable Viability Dye eFluor 780 (eBioscience, Thermo Fisher Scientific) was added to 100 μl unlysed blood to stain dead cells. Then, a cocktail of the following antibodies was added and incubated 30 min at room temperature (RT) in the dark: CD3 Alexa Fluor 488 (BioLegend, San Diego, CA), CD45RA PE (BioLegend), CD127 PE-Cy7 (BioLegend), CD8 APC-Cy7 (BioLegend, San Diego, CA) CD14-CD16-CD19 and CD56 APC-eFluor 780 as dump markers (eBioscience, Thermo Fisher Scientific), CD25 Brilliant Violet 421 (BioLegend, San Diego, CA), and CD4 Brilliant Violet 510 (BioLegend, San Diego, CA). Erythrocytes were lysed by adding 2 ml of BD FACS Lysing Solution (BD Biosciences). After washing, cells were fixed and permeabilized for 30 min at 4 °C and intracellular staining with FOXP3 PerCP‐Cy5.5 (eBioscience Thermo Fisher Scientific) and Ki67 Alexa Fluor 700 (BD Biosciences) was performed for 1 h at RT, using the FOXP3/Transcription Factor Staining Buffer Set according to the manufacturer’s instructions (eBioscience Thermo Fisher Scientific). Data were acquired on an LSR Fortessa cell analyzer (Becton, Dickinson and Company, New Jersey) and analyzed with FlowJo software (version 10.7.1; BD Biosciences). For the gating strategy (Supplementary Fig. 1), lymphocytes were selected based on SSC-A and FSC-A, doublets excluded using FSC-A versus FSC-W and SSC-A versus SSC-W gates. Total Tregs were gated as CD3^+^ CD4^+^ CD127^low^ CD25^high^ live cells staining negative for dump markers identifying non-T-cell lineages (CD14, CD16, CD19 and CD56) and negative for CD8. The Treg population was confirmed to be FOXP3^+^. The subset of activated Tregs (actTregs) was identified as FOXP3^high^ CD45RA^low^ population.

### Clinical data collection

Investigators who were not involved in the eligibility and enrollment phases, prospectively recorded prenatal, perinatal, and postnatal data using a structured and codified data form, from birth, until discharge, transfer to another hospital or death. The sex of the neonates included in the study was defined based on sexual anatomy, in particular after the clinical inspection of external genitalia at birth. Newborns with ambiguous genitalia and/or with external features that differed from the sex established through prenatal testing were excluded. Modalities of the administration of nutrition were prospectively recorded. Diagnosis of NEC was reported in the coded form. Diagnosis of major morbidities of prematurity was performed according to the standard criteria by physicians unaware of the study aims.^5^

### Feeding protocol

Mother milk and preterm formula represented the two available options for enteral nutrition. EN was started, with a minimal enteral feeding (10–20 ml/kg/day divided into four to eight feeds) commenced as soon as the general clinical condition was stable. Between 48 and 96 h, our protocol recommends increasing the feeds of 15–30 ml/kg/day according to birth weight in the absence of FI in the previous 24 h. Donor human milk was not available, in our NICU, during the study period. All subjects were evaluated daily. Aspirate residual from the orogastric tube was measured prior to every feed. Until full enteral feeding (FEF) was reached, PN was administered through central vascular access as previously described.^20^ In the presence of erythematic abdominal wall, absence of bowel sounds or blood in the stools or in aspirates associated with a radiological marker of NEC-Bell stage >I,^4,19^ enteral nutrition was suspended. PN was administered through central vascular access in all subjects to maintain adequate fluid, electrolytes, and nutrient intake until FEF (120 kcal/kg/day) was reached. Total amount of enteral and parenteral fluids was started at 70–100 ml/kg/day and advanced by increments of 20 ml/kg/day until 150– 180 ml/kg/day. Probiotics were not routinely used, in the NICU, during the study period.

### Management of NEC

Supportive medical management of NEC was started promptly as soon as NEC was suspected. Medical management for suspected and confirmed NEC overlap.^21^ Pediatric surgical consultation is advised in every case of suspected NEC. Infants with suspected or confirmed NEC were placed nil per os to allow for bowel rest.^21^ A gastric tube for bowel decompression and monitoring or aspirate was placed. An initial radiograph of the abdomen and left lateral decubitus of cross-table view were obtained to roll out evidence of free air. Serial and positional abdominal radiographs with a frequency of 1–2/day consistent with the suspicion and cadence of advancing clinical disease were obtained, followed by the initial series. A complete blood count, including differences in platelet counts, electrolyte measurement, blood gas, lactate and indices of liver function and coagulation, was performed at least once per day, according to the evolution of the clinical condition. Correction of anemia, thrombocytopenia, electrolyte disturbance, and coagulopathy were performed when necessary. Antimicrobial coverage, broadly targeting gram-negative and anaerobic bacteria, was performed for 7–14 days based on clinical suspicion, confirmation disease, and infants’ clinical course.^21^ Infants who developed NEC stage more than III were an absolute indication for surgical consultation and intervention.

### Statistical analysis

Statistical analysis was performed using Statistical Package for Social Science Software for Microsoft Windows (SPSS Inc, Chicago, IL), version 22.0 and Prism software (version 8, GraphPad). We checked for normality using a Shapiro–Wilk test. The mean and standard deviation or median and interquartile range summarized continuous variables. We used a *χ*^2^ test for categorical variable, *t*-test, Mann–Whitney and Wilcoxon test for paired and unpaired variables. After checking for assumptions, linear regression analysis with a stepwise method was used to study the possible influence of confounding variables (BW, sex as a biological attribute, pH on cord blood, NEC) on Treg proportion at T0. Correlations were assessed with categorical variables by Wilcoxon rank sum tests and with continuous variables by Pearson correlation. We performed a binary regression analysis to study the possible influence of covariates (gestational age (GA), early NE <24 h, breast milk >50% of NE, during the first week of life, Tregs <4%) on the occurrence of NEC. The level of significance for all statistical tests was two-sides (*p* < 0.05). Statistician was blinded to study aims and the patient codes were revealed after statistical analysis.

## Results

We enrolled 58 newborns. During hospitalization, 6 newborns developed NEC stage II; thus, 52 served as controls. Among controls, we identified 2 Groups: (i) group 1 included 23 neonates with the occurrence of FI (FI controls); and (ii) group 2 included 29 neonates without FI (FeedTol controls) (Table 1). The main clinical characteristics of participating cases and controls are summarized in Table 1. Baseline clinical characteristics were similar between cases and controls. The rate of male and female neonates was similar between cases and controls; the rate of female neonates is reported in Table 1. The GA was significantly higher in FeedTol controls compared with NEC cases (Table 1). The duration of PN and the timing of FEF were significantly longer in cases compared either with controls (overall) or with FeedTol controls, but not when compared with FI controls (Table 1).

**Table 1:** Baseline clinical characteristics of the study population.

	Cases	Controls		
	NEC	Overall	FI	FT
Number	6	52	23	29
Prenatal characteristics				
GA, weeks	31.17 ± 1.47	32.06 ± 2.43	30.70 ± 2.85	33.14 ± 1.30**
GA ≤28, weeks	1 (16.7)	5 (9.6)	5 (9.6)	0 (0.0)
Twins, *N* (%)	3 (50)	36 (69.2)	16 (69.5)	20 (60.9)
IUGR, *N* (%)	2 (33.3)	11 (21.6)	6 (27.3)	5 (17.2)
Cesarean section, *N* (%)	5 (83.3)	49 (94.2)	23 (100)	26 (89.7)
Mother’s age >35 years old, *N* (%)	2 (40)	17 (34)	8 (38.1)	9 (31.0)
Hypertension, *N* (%)	0 (0)	5 (9.8)	3 (13.6)	2 (6.9)
Hypothyroidism, *N* (%)	0 (0)	10 (19.6)	5 (22.7)	5 (17.2)
Gestational diabetes, *N* (%)	0 (0)	11 (21.6)	7 (31.8)	4 (13.8)
Antenatal corticosteroids^a^, *N* (%)	4 (80)	36 (72)	16 (72.7)	20 (71.4)
Uterine arteries flowmetry alteration, *N* (%)	0 (0)	6 (12)	3 (14.3)	3 (10.3)
Postnatal characteristics				
Female sex, *N* (%)	4 (66.7)	21 (40.4)	7 (30.4)	14 (48.3)
Small for gestational age, *N* (%)	1 (16.7)	11 (21.2)	5 (21.7)	6 (20.7)
BW, *N* (%)	1310 ± 681	1650 ± 503	1413 ± 485	1837 ± 440**
BW ≤1000, g	2 (33.3)	6 (75)	5 (21.7)	1 (3.4)
pH on cord blood	7.258 ± 0.064	7.270 ± 0.776	7.254 ± 0.101	7.281 ± 0.055
Start of EN, DOL	3.60 ± 4.099	2.02 ± 6.629	3.95 ± 9.84	0.55 ± 0.78**
Duration of PN, DOL	25.00 ± 16.73*	10.17 ± 15.47	20.48 ± 18.59	2.00 ± 2.87**
Full enteral feeding, DOL	26.50 ± 16.42*	11.53 ± 15.57	22.45 ± 18.56	3.24 ± 3.12**
Breast milk from 0 to 7 DOL, ml/die	92.50 ± 171.9	99.36 ± 191.5	54.67 ± 147.1	132.89 ± 215.48

Data were expressed as mean ± standard deviation when not specified.

*FI* feeding intolerance, *FT* feeding tolerance, *GA* gestational age, *IUGR* intrauterine growth restriction, *BW* birth weight.

*p < 0.001 NEC versus controls (overall); **p < 0.001 NEC versus FT.

^a^An intramuscular steroids cycle in two doses of 12 mg over a 24-h period.

Mortality rate was similar between cases and controls overall (0.0% vs. 1.9%; *p* = 0.897) and between cases and FI controls (0.0% vs. 4.3%; *p* = 0.793). There was no significant difference in length of hospital stay (days) between cases and controls overall (55.2 vs. 43.35; *p* = 0.364) or between NEC and FI controls (55.2 vs. 58.00; *p* = 0.868).

We performed a correlation between Treg percentage at three time points and FEF at both 7 and 14 days. We did not find a statically significant correlation (FEF at 7 days *p* = 0.272; FEF at 14 days *p* = 0.102)

**Fig. 1:**
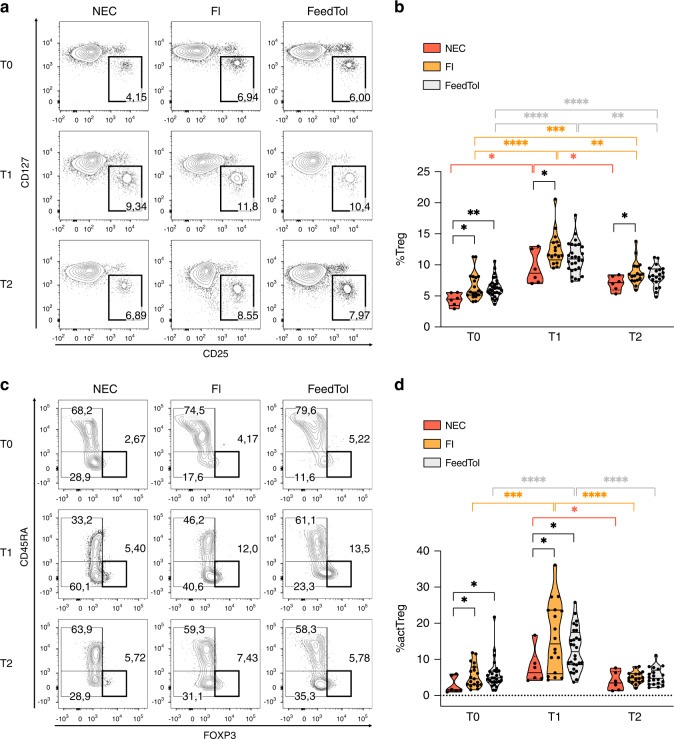
Neonates who develop NEC are characterized by a lower frequency of Tregs and actTregs as compared to control neonates with (FI) or without (FeedTol) feeding intolerance, at early and late time points post birth. **a**, **b** Representative plots (**a**) and cumulative analysis (**b**) showing the percentages of Tregs (CD127^low^ CD25^high^) in gated CD4^+^ cells, estimated at different time points in peripheral blood of preterm neonates who develop NEC (*n* = 6) and in controls with feeding intolerance (FI, *n* = 19) or tolerance (FeedTol, *n* = 28). Blood samples were collected from each neonate at 0–3 (T0), 7–10 (T1), or 28–31 (T2) days post birth. **c**, **d** Representative plots (**c**) and cumulative analysis (**d**) showing the percentages of CD45RA^low^FOXP3^high^ actTregs (heavy thickness square) in gated Tregs (identified as CD127^low^ CD25^high^ CD4 T cells), in the same samples as above. Numbers indicate the percentages of each subset. *p < 0.05, **p < 0.01, by Mann–Whitney test, between subgroups. *p < 0.05, **p < 0.01, ***p < 0.001, ****p < 0.0001, by Wilcoxon matched-paired test, between time points within each subgroup. In each analysis, only data from neonates whose samples were available at least at both T0 and T1 were included.

To understand if the Treg population may play a role in NEC occurrence, we performed a multiparametric flow cytometry analysis in the peripheral blood of the neonates whose samples were collected at least at both T0 and T1, to quantify the frequency of total Tregs (identified as CD127^low^ CD25^high^ in gated CD4 T cells) and of the highly suppressive actTreg subset (identified as FOXP3^hi^ CD45RA^low^ in gated CD127^low^ CD25^high^ Tregs). In line with Hayakawa et al.,^13^ we found a significant increase in Treg frequency from T0 to T1, which almost returned to the baseline at T2, in NEC cases as well as in controls. However, as shown in Fig. 1a, b and Supplementary Fig. 2, at T0 we observed a significantly lower frequency of Tregs in NEC cases compared with both FI and FeedTol controls. Moreover, a statistically significant difference in Treg frequency was maintained in the later time points (T1 and T2) between NEC cases and FI control group (Fig. 1). The percentage of Teff, identified as CD127^+^ CD25^+^ CD4 T cells, or the proportion of activated (CD45RA^-^) cells in the Teff gate, did not vary significantly between samples or time points (Supplementary Fig. 3). Interestingly, at T0 and T1, we also found a significantly lower percentage of actTregs in neonates with NEC compared to the other two groups (FI controls and FeedTol controls), (Fig. 1c, d). Accordingly, the actTreg frequency increased significantly from T0 to T1 in both FI and FeedTol controls, but not in NEC cases (Fig. 1d). Conversely, we did not observe any difference in total Treg and actTreg frequencies between FI and FeedTol control groups, at any time point (Fig. 1a–d).

The linear regression analysis revealed that the proportion of Tregs at T0 was significantly (*p* = 0.010) related to the occurrence of NEC in a multivariate model (Table 2). As shown in Table 3, the binary logistic regression analysis reported that the occurrence of NEC in preterm neonates was independently influenced by Treg frequency at birth.

**Table 2:** Linear regression analysis to evaluate the influence of covariates on the proportion of Tregs at T0.

Variables	*ß*	Wald	*p* value
GA	–0.195	–1.421	0.162
Sex	0.004	0.029	0.977
PH on cord blood	–0.067	–0.488	0.628
NEC	–0.372	–2.674	0.010*

*GA* gestational age, *NEC* necrotizing enterocolitis.

*p < 0.05.

**Table 3:** Binary logistic regression to evaluate the influence of variables on the NEC development.

Variables	*ß*	SE	Wald	*p* value	Odds ratio (OR)	95 CI for OR	
						Lower	Upper
GA	2.178	1.858	1.373	0.241	8.828	0.231	337.0
Early NE^a^	–0393	1.380	0.081	0.776	0.675	0.045	10.08
Breast milk^b^	1.564	1.645	0.904	0.342	4.776	0.190	120.0
Tregs at T0^c^	2.989	1.447	4.625	0.039*	19.860	1.164	338.74

*GA* gestational age, *NE* enteral nutrition.

*p < 0.05

^a^NE administered <24 h of life.

^b^Brest milk >50% of total enteral nutrition.

^c^Tregs (Treg <4%).

**Fig. 2:**
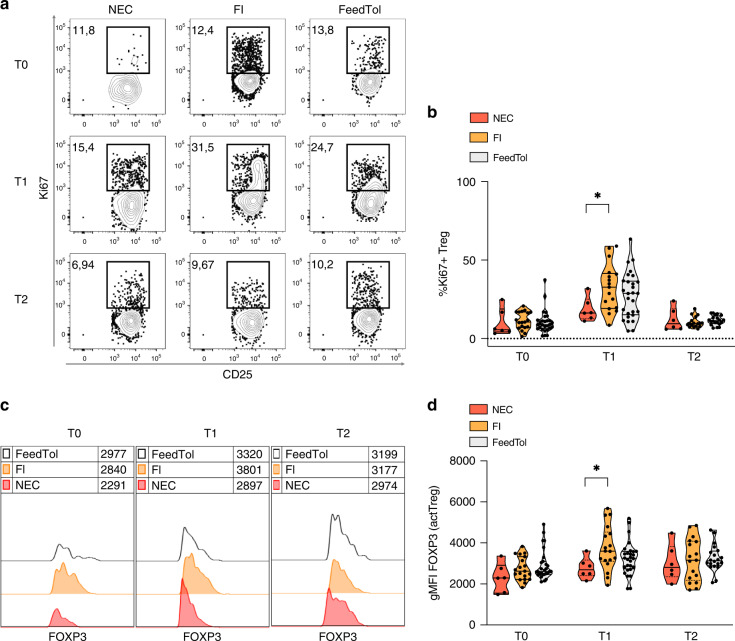
In neonates with NEC, Tregs are less proliferative and actTregs express a lower level of FOXP3 compared to controls with feeding intolerance, at 7–10 days post birth. **a**, **b** Representative plots (**a**) and cumulative analysis (**b**) showing the percentages of Ki67^+^ cells in gated CD127^low^ CD25^high^ Tregs from the peripheral blood of preterm neonates with NEC (*n* = 6), feeding intolerance (FI, *n* = 19) or feeding tolerance (FeedTol, *n* = 28), collected at 0–3 (T0), 7–10 (T1), or 28–31 (T2) days post birth. Numbers indicate the percentages of Ki67^+^ cells. **c**, **d** Representative histograms (**c**) and cumulative analysis (**d**) showing FOXP3 expression in actTreg population in the same samples as above. Numbers in the histogram plots indicate the gMFI (geometric mean fluorescence intensity) of FOXP3 expression in gated CD45RA^low^ FOXP3^high^ actTregs in the neonate subgroups as indicated in the legends. *p < 0.05, by Mann–Whitney test. In each analysis, only data from neonates whose samples were available at least at both T0 and T1 were included.

To verify if the low Treg percentage in neonates with NEC could be attributed to a defect in Treg proliferation, we analyzed the frequency of Tregs that expressed Ki67, a marker of cell cycling. We found a significantly lower frequency of Ki67^+^ Tregs in NEC cases compared to FI controls at T1; a similar trend was also observed when we compared NEC cases with FeedTol controls at the same time point (Fig. 2a, b). Finally, we analyzed the expression intensity of FOXP3 in actTregs (identified as indicated above). At T1, in neonates with NEC, actTregs expressed a significantly lower level of FOXP3 (in terms of geometric mean fluorescence intensity) compared to actTregs in FI control group, and the same trend was observed at T1 also compared to FeedTol controls (Fig. 2c, d). In throughout the analysis, we verified the drop in Tregs only in proportions, the overall Treg numbers were not accessed.

## Discussion

Results of our study suggest that Treg frequency and features in the peripheral blood of preterm neonates may be useful in identifying neonates at high risk of developing NEC early in life when clinical signs of NEC were not already evident.

Neonates who developed NEC afterward displayed a significantly lower frequency of Tregs and actTregs as compared to neonates without the disease; interestingly, neonates with NEC showed a lower proportion of both cell populations as compared to neonates with FI, at both early and late time points post birth.

Previous studies that investigated the role of mucosal Tregs in the pathogenesis of NEC mainly in animal models supported the critical role of Tregs in maintaining intestinal immune homeostasis.^22,23^ These authors hypothesized that necrosis of the intestine is a consequence of a deregulated inflammatory response that, in turn, depends on diminished tolerogenic Tregs.^15,24^ He et al., in a more recent case–control study, found that the frequency of Tregs in the mononuclear cells of lamina propria was decreased in NEC mice compared to mice without the disease.^24^ Previous clinical studies in newborns suggested a role of Tregs in the pathogenesis of NEC;^6,13^ however, the power of Tregs in predicting NEC development is largely undefined. A randomized controlled trial investigated the frequency of Tregs in preterm newborns who received bovine colostrum.^25^ They found that newborns receiving bovine colostrum during the first 2 weeks of life showed an increased percentage of blood Tregs. The authors observed a positive trend for reduction of sepsis severity and mortality in the bovine colostrum group; however, the relation between Tregs and NEC occurrence was not specifically investigated.^25^

In a clinical case–control study, the authors analyzed by flow cytometry the percentage of mucosal Tregs in the lamina propria of surgical patients with NEC versus non-NEC surgical controls, and they found a significantly higher frequency of Tregs in the control group than in NEC patients.^24^ Moreover, in ileal tissues of NEC patients, they observed a gene expression profile characterized by the increased expression of inflammatory cytokines and lower expression of genes related to Treg induction and function compared to control samples.^24^ However, the study was performed on newborns with different GA; moreover, Tregs were studied when the detrimental effects of inflammation were already in place. Similar results were obtained, recently, by Weitkamp et al., who investigated Treg and effector CD4 and CD8 T cell composition in surgical tissue specimens of preterm newborns with NEC compared with age-matched controls (i.e., resections for spontaneous intestinal perforation, congenital intestinal atresia, small bowel obstruction, gastroschisis with bowel necrosis and tissue from re-anastomoses for various surgical indications).^6^ They found a reduction of Tregs to effector T cell proportions in NEC versus non-NEC lamina propria and suggested that these decreased ratios might contribute to the pathology and severity of the disease. Besides, mucosal Tregs in NEC samples showed less evidence of activation and gut homing. However, those results are limited to preterm neonates with surgical NEC. Thus, the sample collection was performed when the inflammation-mediated damages in the intestinal tract resulted already in necrosis. Therefore, the impairment of Tregs, observed in that study, may represent an epiphenomenon of necrosis rather than a predisposing factor. A more recent case–control study observed that the frequency of blood Tregs was significantly lower in preterm newborns affected by NEC.^17^ In this study, authors evaluated the function of Tregs, and they observed a significantly lower suppression capacity of Tregs in cases compared with controls.

Recent observational study examined the fluctuation of the number and composition of the Treg population in newborns.^13^ Authors found that Tregs were increased in the early neonatal period, specifically at 7 days of life; however, the direct relation between Tregs and the occurrence of NEC was not investigated. It has been described that after birth, during perinatal life, there is a transient increase of Tregs in preterm human infants.^13,26^ In particular, the increase of Tregs peaks at 7–10 days after birth; moreover, in parallel with this Treg peak, an increased frequency of Ki67^+^ proliferating cell subsets was observed.^13,26^ In our study, we characterized more in detail some phenotypical features of Treg and actTreg populations, and our data suggest that, at a very early time point (i.e., 0–3 days after birth), the analysis of Treg and actTreg percentage could provide a predictive factor of NEC development in preterm neonates. Our analysis could not include the characterization of peripherally induced Tregs, developing in the intestinal mucosa mostly in response to bacterial antigens. However, intestinal Tregs also comprise thymic Tregs that, in the perinatal life, have extensively proliferated, differentiated into tissue-Tregs, and then colonized several organs.^27,28^ Therefore, the frequency of circulating Tregs may reflect the extent of the perinatal proliferation that precedes tissue-Treg differentiation. Indeed, these two events may be tightly linked since tissue-Treg precursors have been found to be highly proliferative in a mouse model.^28^ Our findings indicated that, besides the frequency, also the phenotypical characteristics of Treg and actTreg populations may be crucial in predicting NEC development. Indeed, in neonates with NEC, Tregs are less proliferative and actTregs express a lower level of FOXP3 compared to neonates with FI at T1 (i.e., 7–10 days post birth). We could not perform a suppression assay ex vivo, due to the extremely low amount of blood that did not allow the isolation of sufficient Treg numbers, However, the lower expression intensity of FOXP3 could be linked to weaker stability and potency of actTregs in NEC samples. Therefore, early in life, the reduced proliferation of Tregs and the lower FOXP3 expression in actTregs may allow us to identify the preterm neonate with FI in which NEC will occur before disease onset.

The analysis at T0 includes data from neonates at 0–3 days of life, which is a very early time point during which babies face rapid and individual changes due to the transfer from the intrauterine to the extrauterine environment. For this reason, the timing at which Tregs start to proliferate might be variable, and this could affect the measurement at this time point. Hence, in the small NEC cohort, the variability in the frequency of Ki67+ cells did not allow to highlight any differences compared to the control cohorts. On the contrary, at T1, the Treg proliferation rate was well established in all cohorts, and this allowed us to reveal significant differences in the frequency of proliferating cells.

Pang et al. designed a case–control study to better identify the reason underlying the reduced Treg proportion in preterm newborns with NEC. They studied the role of monocytes in promoting Treg differentiation in preterm neonates with and without NEC. They found that monocytes in NEC patients displayed a proinflammatory profile that could suppress Treg induction, thus preventing the activity of Tregs. Nevertheless, the authors did not focus on the direct role of Tregs in NEC development.^16^ Even in that study, the timing of sampling was performed when the disease was clearly ongoing. In our study, we performed the sample collection at birth, prior to the onset of signs and symptoms of NEC, and thus, prior to the actual development of the deregulated immune response potentially leading to necrosis. Hence, we provide the first evidence that an early reduction of Treg frequency and proliferation predisposes preterm newborns to the risk of developing NEC. It is worth mentioning that the significant difference in Treg percentages, observed between NEC newborns compared with neonates with symptoms of FI, underlines the promising role of the early identification of this immune status on clinical outcome.

The results of our study should be interpreted considering some limitations. Firstly, the association between Tregs at birth and NEC may be related to the effects of chance (random error), bias or confounding factors. To limit the risk of biases, we verified the effects on NEC occurrence after correcting for confounding variables. To limit selection and spectrum bias, strict inclusion and exclusion criteria were adopted, and physicians were informed of the study methodology in several meetings. In addition, we considered all the eligible patients and enrolled both cases and controls consecutively. To minimize information bias, clinical data were collected by researchers different from those who measured Treg population in blood samples. Researchers who collected clinical data were unaware of the results of Treg analyses. As the diagnosis of NEC is mainly based on clinical ground, the risk of misclassification bias was high: in order to improve this aspect, classification of enrolled newborns as cases or controls was confirmed after an agreement between two researchers (G.T. and M.D.C.). Analysis of Tregs was performed by a researcher blinded regarding clinical information. Finally, statistical analysis was performed by a statistician blinded to the study aims. The study results should be interpreted considering the small sample size. We classified as cases preterms with signs and symptoms suggestive of NEC.

The reduced number of newborns, particularly those included in NEC cohort, limits the generalizability of the results. Furthermore, the drop in Tregs was only in proportions and overall Treg numbers were not accessed. The overlap between the proportions of Tregs in those with NEC and those with FI is very large; designing a study with an increased number of neonates may help to reduce the dispersion of values. Infants born extremely preterm are at much higher risk for NEC and their Treg values are unknown. The overall age of the NEC cohort is more than 30 weeks, only one infant of 28 weeks of GA was included in the cohort. Further studies, with a longer study period, including newborns with lower GA, which are at higher risk of developing NEC, are advocated.

In conclusion, our study suggests that the occurrence of NEC in preterm neonates could depend on the proportion of Treg at birth. Currently, there has been no study to demonstrate a direct effect of Treg features in inducing the development of NEC. Insights into the role of Tregs in mediating the NEC pathogenesis would have an impact in understanding this devastating disease. This study encourages further research on the clinical role of Tregs in order to translate in the nutritional and medical management of preterm newborns at risk of NEC the findings derived from immunopathogenesis studies.

### Supplementary information

Supplementary Figure 1

Supplementary Figure 2

Supplementary Figure 3

## Data Availability

The datasets analyzed during the current study are available from the corresponding author on reasonable request.
