# Supplementary figures and images for: Reduction in regulatory T cells in preterm newborns is associated with necrotizing enterocolitis
Source: Pediatr Res. 2023 Jun 21;94(5):1789–96. doi: 10.1038/s41390-023-02658-3 (PMC10624602; doi:10.1038/s41390-023-02658-3)

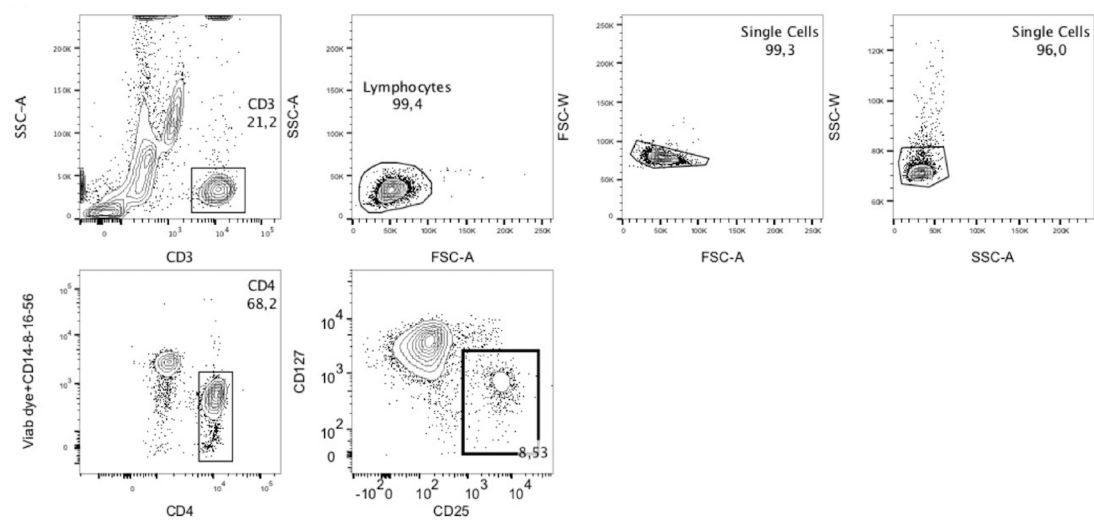

Supplemental Figure 1

Supplement: Supplementary file 1 — Supplementary Figure 1 [file 41390_2023_2658_MOESM1_ESM.pdf]

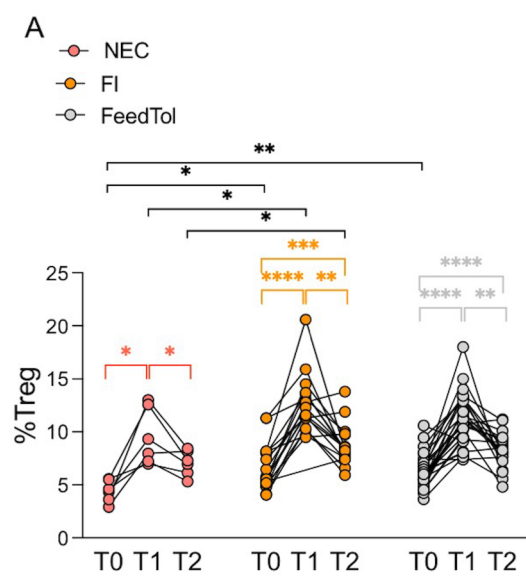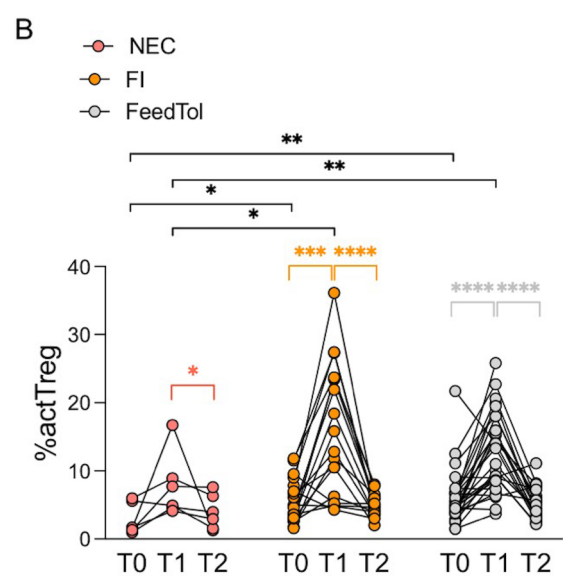

Supplemental Figure 2

Supplement: Supplementary file 2 — Supplementary Figure 2 [file 41390_2023_2658_MOESM2_ESM.pdf]

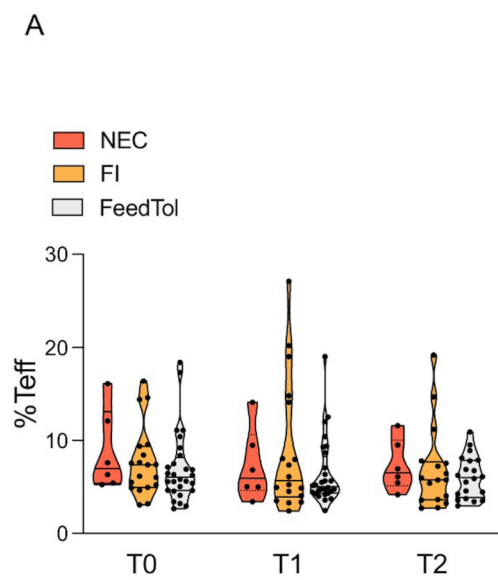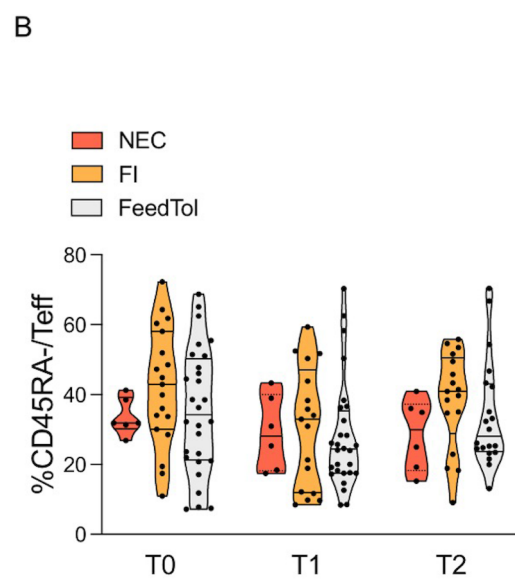

Supplemental Figure 3

Supplement: Supplementary file 3 — Supplementary Figure 3 [file 41390_2023_2658_MOESM3_ESM.pdf]
